# The aldehyde hypothesis: metabolic intermediates as antimicrobial effectors

**DOI:** 10.1098/rsob.220010

**Published:** 2022-04-13

**Authors:** K. Heran Darwin, Sarah A. Stanley

**Affiliations:** ^1^ Department of Microbiology, New York University Grossman School of Medicine, New York, NY, USA; ^2^ Department of Molecular and Cell Biology, Division of Immunology and Pathogenesis, University of California, Berkeley, CA, USA; ^3^ Division of Infectious Disease and Vaccinology, School of Public Health, University of California, Berkeley, CA, USA

**Keywords:** aldehydes, *Mycobacterium tuberculosis*, nitric oxide, interferon-gamma, macrophages, innate immunity

## Abstract

There are many reactive intermediates found in metabolic pathways. Could these potentially toxic molecules be exploited for an organism's benefit? We propose that during certain microbial infections, the production of inherently reactive aldehydes by an infected host is a previously unappreciated innate immune defence mechanism. While there has been a significant focus on the effects of aldehydes on mammalian physiology, the idea that they might be exploited or purposefully induced to kill pathogens is new. Given that aldehydes are made as parts of metabolic programmes that accompany immune cell activation by the cytokine interferon-gamma (IFN-γ) during infections, we hypothesize that aldehydes are among the arsenal of IFN-γ-inducible effectors needed for pathogen control.

## Graphical abstract

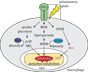

## 1. Introduction

All forms of life are susceptible to infection, whether it is by viruses, bacteria, fungi or parasites. Humans have evolved highly complex defence mechanisms against virtually all forms of infectious agents, and these mechanisms may be tailored to their targets. For example, while antibodies are critical for controlling many viral infections, they are often ineffective against pathogens that live within a host's cells. Unicellular microbes, either bacterial or eukaryotic, are often captured by phagocytes such as neutrophils and macrophages. These soldiers of the immune system have numerous mechanisms to inhibit or eliminate microbial growth, including the production of pore-forming peptides, reactive oxygen and nitrogen species; the import and export of transition metals and exposure to destructive enzymes such as proteases and lipases (reviewed in [1]).

The human-specific pathogen *Mycobacterium tuberculosis* appears to frequently evade sterilization from infected hosts. As a consequence, and by some estimates, *M. tuberculosis* has infected nearly one-third of the world's population, with most individuals controlling their infections without medical intervention. While this number has been recently debated [2], it is clear that *M. tuberculosis* kills about 1.7 million people annually, making it a leading cause of death by a single infectious agent [3]. Activation of macrophages by the cytokine interferon gamma (IFN-γ) is crucial for control of mycobacterial infections [4]. Historically, the importance of IFN-γ has been attributed to its ability to induce the expression of nitric oxide synthase (iNOS), and mouse studies have shown an essential role for nitric oxide (NO) in robustly controlling *M. tuberculosis* growth [5]. While these data provide compelling evidence of its importance, it has been challenging to demonstrate that NO has a definitive role in controlling *M. tuberculosis* in humans, given the paucity of humans defective for iNOS and the lack of a robust *in vitro* infection model using human cells. Furthermore, several animal studies have suggested a role for NO in the regulation of inflammation that is separate from its putative antimicrobial activity [6,7]. In addition to NO, copper (Cu) may also help control *M. tuberculosis* infections [8–11]. *In vitro* macrophage data show the mobilization of the essential Cu transporter ATP7a from the Golgi complex to bacteria-containing vacuoles by IFN-γ, suggesting mammals have evolved to weaponize this potentially toxic metal during infections [12,13]. Supporting this hypothesis is the observation that Cu supplementation can dramatically reduce *M. tuberculosis* burden in animals [8]. Despite these advances in our knowledge of host defences, gaps remain in our understanding of the antimicrobial effectors used by macrophages to control *M. tuberculosis* infection*.* In this review, we outline evidence to suggest host-derived aldehydes contribute to controlling *M. tuberculosis* and possibly other pathogens, and represent a newly appreciated weapon in the innate immunity arsenal.

## 2. *Mycobacterium tuberculosis* is highly susceptible to changes in its aldehyde metabolism

Several studies have indicated that *M. tuberculosis* is highly susceptible to aldehydes. Disruptions in various mycobacterial metabolic pathways can result in increased intracellular aldehydes, reducing bacterial survival under *in vitro* and *in vivo* conditions. In one study, a screen for compounds against *M. tuberculosis* identified a class of pyrimidine-imidazoles (PIs) that strongly inhibit growth, possibly by increasing the amount of several intracellular aldehydes [14]. Pethe *et al.* [14] found that PIs cause *M. tuberculosis* to accumulate glycerol phosphate while depleting adenosine triphosphate (ATP). Glycerol phosphate can enter the glycolytic pathway and be converted into dihydroxyacetone phosphate (DHAP), which isomerizes into glyceraldehyde-3-phosphate (GAP). Furthermore methylglyoxal (MG), also known as pyruvaldehyde, can spontaneously form from DHAP and is well known to be toxic. Interestingly, the authors of this study demonstrated that the exogenous addition of GAP, DHAP and MG to cultures of *M. tuberculosis* is toxic, demonstrating the direct antimicrobial activity of these aldehydes or aldehyde precursor (DHAP). Further supporting the authors' hypothesis that the accumulation of glycerol phosphate or its downstream products leads to toxicity, a genetic screen for *M. tuberculosis* mutants resistant to the PIs identified mutations in *glpK* (glycerol kinase, Rv3696c). Because GlpK phosphorylates glycerol into glycerol-3-phosphate, its disruption could potentially prevent the accumulation of GAP, DHAP and MG, thereby reducing overall aldehyde levels in bacteria grown in glycerol.

Given that several aldehydes possibly accumulate in *M. tuberculosis* treated with PIs, Pethe *et al.* [14] proposed that the PIs targeted one or more aldehyde detoxification enzymes. To test this hypothesis, the authors pulled down proteins that interact with PIs and identified four putative MG detoxification enzymes. Ectopic production of one of these proteins Rv0577 improves bacterial growth in glycerol and provides some resistance to two of the PI compounds, suggesting it has a role in aldehyde detoxification. A caveat to these studies is that the ability of the PIs to directly inhibit the activities of one or more of the identified enzymes was not tested. Interestingly, GlpK was also the focus of two recent independent studies that determined glycerol metabolism *in vitro* or *in vivo* makes *M. tuberculosis* more susceptible to several front-line tuberculosis (TB) drugs. The Alland group found that naturally occurring frame shift mutations in *glpK* appear to be selected for in *M. tuberculosis* clinical isolates, while the Sassetti laboratory used a transposon sequencing screen to find mutants that provided increased fitness in antibiotic-treated mice [15,16]. Both groups found defects in glycerol metabolism made bacteria more tolerant to several different antibiotics. While neither study determined how disruption of *glpK* protected *M. tuberculosis*, Sassetti and co-workers [7] proposed that MG or other products of glycerol metabolism could increase antibiotic efficacy. It is tempting to speculate that aldehydes like MG or GAP could help potentiate the activity of one or more drugs against *M. tuberculosis* and other microbes.

In addition to interference with glycerol metabolism, several other laboratories noted that disruption of certain reactions around the tricarboxylic acid cycle or glyoxylate shunt result in the accumulation of aldehydes in *M. tuberculosis*. In a study by Puckett *et al*., deletion of the malate synthase gene (Rv1837c) resulted in the accumulation of glyoxylate, making bacteria highly attenuated for *in vitro* and *in vivo* growth [17]. Similarly, a study by the Nathan laboratory determined that disruption of 2-hydroxy-3-oxoadipate synthase (HOAS, Rv1248c), the E1 component of *α*-ketoglutarate dehydrogenase, results in the accumulation of succinate semialdehyde and glyoxylate [18]. A mutant defective in HOAS is highly sensitive to NO, although the connection between HOAS and NO sensitivity is unclear. These studies aligned well with a report from the Darwin laboratory; work in the Nathan laboratory previously determined that *M. tuberculosis* mutants defective in proteasome activity are highly sensitive to NO, although it was unknown how protein degradation was linked to this phenotype [19]. Remarkably, a genetic suppressor screen found that a single proteasome substrate called Log (lonely guy, Rv1205) is responsible for the NO sensitivity of proteasomal mutants. Log catalyses the final step in the production of signalling molecules known as cytokinins, which can be broken down into adenine and various aldehydes. Indeed, cytokinin-associated aldehydes are sufficient to sensitize wild-type *M. tuberculosis* to NO [19]. Interestingly, the Darwin laboratory also found that a mutation in *glpK* suppresses the NO-sensitive phenotype of a proteasome mutant, further supporting a link between aldehyde accumulation and NO sensitivity [19]. Collectively, these data strongly suggest *M. tuberculosis* must tightly regulate its intracellular aldehyde levels to prevent toxicity from aldehydes alone, as well as mitigate sensitization of the bacteria to NO and possibly other chemicals by the aldehydes. However, more research is required to clearly establish mechanistic links between intracellular aldehyde accumulation and bacterial fitness.

## 3. Phagocytes produce a variety of aldehydes

There are abundant data that show macrophages and neutrophils produce copious amounts of aldehydes. For example, Heinecke and co-workers [20] showed that activated neutrophils can oxidize almost any amino acid into an aldehyde. This process requires myeloperoxidase, which is released from granules in neutrophils during infections, as well as by hydrogen peroxide (H_2_O_2_) produced by an oxidative burst in phagocytes. Reactive oxygen species (ROS) such as H_2_O_2_ can also oxidize lipids into highly toxic lipid aldehydes including 4-hydroxynonenal (4-HNE) and malondialdehyde (MDA).

The activation of macrophages and other cell types to produce ROS and mobilize other antimicrobial effectors requires IFN-γ, which is one of the most important cytokines needed for the control of *M. tuberculosis* [4]. The downstream effectors of IFN-γ signalling needed to control bacterial growth are, however, incompletely understood. Recently, the Stanley and other laboratories have shown that IFN-γ strongly induces aerobic glycolysis during *M. tuberculosis* infections of mouse or human macrophages [21–23]. Aerobic glycolysis, also known as the Warburg effect, is a pathway by which cells rely exclusively on glycolysis for the production of ATP, even in the presence of oxygen. Several laboratories have reported the observation that a ‘Warburg-like' effect is induced in M1 macrophages in various infection settings and that this metabolic programme supports host control of infections with *M. tuberculosis*, *Legionella pneumophila* and *Streptococcus pneumoniae* [22,24,25]. Furthermore, the IFN-γ responsive transcription factor HIF-1*α* requires aerobic glycolysis for its stabilization in macrophages during immune activation. The fact that HIF-1*α* is essential for control of *M. tuberculosis* as well as other pathogens, strongly suggests that aerobic glycolysis is an integral component of a host-protective immunometabolic programme [21,22,25]. Further supporting this idea, the ability of *Salmonella* Typhimurium and *Brucella abortus* to establish chronic infection in M2 macrophages, which rely on oxidative metabolism rather than aerobic glycolysis, has been linked to excess glucose levels. It has also been proposed that the induction of aerobic glycolysis is exploited by various pathogens for their benefit, for example, by providing nutrients like lactate or lipids that benefit bacterial growth (reviewed in [26]). However, given that glycolytic intermediates or side-products are aldehydes in mammals just as they are in bacteria, we propose that the induction of glycolysis is a *host*-beneficial adaptation with respect to *M. tuberculosis* and possibly other infections.

## 4. The aldehyde hypothesis

Based on data from the Stanley laboratory, we took a closer look at glycolysis as a pathway that generates aldehydes to contribute to pathogen control downstream of IFN-γ activation. Estimates of GAP and other glycolytic intermediate concentrations in cells range from 15 µM to 1.5 mM, depending on the metabolic state of the cell [27,28]. Relevantly, *M. tuberculosis*-infected macrophages release up to 1.6 mM MG into culture supernatants [29]. At the time of this study, the authors hypothesized that the mycobacteria produce MG to kill macrophages; however, it is more likely that macrophages produce MG in response to infection. Indeed, even sterile immune activation with lipopolysaccharide results in high levels of MG production in macrophages [30–33].

We also hypothesize that the decreased detoxification of aldehydes could benefit a host during infections. A substantial proportion of the world's population of Asian descent harbour a specific loss-of-function mutation in aldehyde dehydrogenase 2 (ALDH2), which greatly reduces its activity. The mutant allele is known as ALDH2*2 (rs671), which has a codon 487 glutamate to lysine substitution. Hetero- or homozygous carriers of ALDH2*2 who drink alcohol accumulate acetylaldehyde, the toxicity of which results in a characteristic facial flushing response. Given that the ALDH2*2 allele incurs deleterious effects even in the absence of alcohol consumption, it appears that there was a strong selective pressure that maintained this mutation in humans. Shin and co-workers [34] performed an association analysis among a cohort of Korean men with various disease states and the ALDH2*2 allele. Strikingly, the only significant correlation they observed was individuals with TB were less likely to have the mutant allele. Much like the mutation that causes sickle cell anaemia protects against malaria in Africa, it is possible that the proliferation of the ALDH2*2 allele served an analogous function against *M. tuberculosis* infections in Asia.

The authors of this study presumed a healthier lifestyle caused by decreased alcohol consumption in individuals with inactive ALDH2 contributed to more resistance to acquiring TB. While alcohol abuse is linked to poor TB outcomes, the precise reasons are unknown and may have either to do with immune fitness, antibiotic regime compliance or a combination of both [35]. Given that it is estimated the ALHD2*2 allele arose approximately 3000 yr ago [36], it is impossible to know if alcohol consumption played a role in TB epidemiology at that time. Perhaps another compelling argument for a protective effect of aldehydes conferred by the ALDH2*2 allele is the appearance of the so-called hypervirulent ‘Beijing' or ‘W' *M. tuberculosis* strains. This lineage appears to have established in China between 2000 and 7000 yr ago [37,38]. While the mechanisms (or definitions) of bacterial hypervirulence are somewhat unclear, these strains have resulted in several outbreaks outside of China, suggesting that they are more transmissible or virulent. We speculate that these strains evolved to be more transmissible within a population with higher intrinsic resistance to *M. tuberculosis*, either due to increased resistance to aldehydes or by reducing the induction of aldehyde-generating pathways by infected cells.

In addition to their anti-pathogen activity, aldehydes are also reactive with and damaging to self. Thus, humans have evolved to make numerous enzymes that convert aldehydes into less harmful molecules. The glyoxalase system in humans, consisting of the enzymes glyoxalase 1 (GLO1), glyoxalase 2 (GLO2) and reduced glutathione, detoxifies MG into d-lactate and is present in all mammalian cells. Modulation of MG levels by GLO1 is thought to influence the pathogenesis of numerous diseases including diabetes, atherosclerosis and neurodegenerative disorders, among others [39]. MG levels are high during sepsis, a hyper-inflammatory state usually caused by bacterial infection [32]. During inflammation, high levels of ROS are produced. ROS can directly suppress GLO1 function [39], and because ROS detoxification also requires glutathione, it can compete with the glyoxalase system. It is therefore possible that during infection, the production of ROS could lead not only to the reactive aldehydes 4-HNE and MDA but also to increased levels of MG. It would be interesting to test whether genetic or pharmacological inhibition of GLO1 could lead to enhanced control of bacterial infection.

## 5. A tuberculosis drug in hand?

Disulfiram (DSF) or antabuse was developed over 50 yr ago to treat alcohol abuse and works by inhibiting ALDH2, mimicking the ALDH2*2 mutation in humans [40]. More recently DSF was identified in a screen to repurpose existing clinical drugs for use in TB patients. The treatment of *M. tuberculosis*-infected mice with DSF results in a significant reduction in bacterial burden, comparable to treatment with the antibiotic rifampin [41]. Because the screen that identified DSF activity against *M. tuberculosis* was performed in the absence of mammalian cells, the authors of this study did not assume that the inhibition of ALDH2 was linked to the anti-tubercular activity of DSF in mice. In an effort to understand the mechanism of action of DSF, Wolschendorf and co-workers [42] found that in acidic or Cu-rich conditions, DSF breaks down into diethyldithiocarbamate (DETC), which complexes with Cu ions. DETC-Cu complexes can enter *M. tuberculosis* bacilli, resulting in bacterial death. Thus, it is presumed that DSF directly kills *M. tuberculosis in vivo* in a Cu-dependent manner. By contrast, DSF does not use Cu to inactivate ALDH2 activity and instead is likely to catalyse the formation of a stable intramolecular disulfide bond between two active site cysteines in ALDH2 [43].

We do not know if DSF is readily converted to DETC *in vivo* or if there is enough bioavailable Cu available to complex with DETC if formed. These observations thus question a Cu-dependent anti-mycobacterial activity of DSF *in vivo*. We therefore hypothesize the inhibition of ALDH2 and concomitant aldehyde accumulation from various metabolic pathways including inflammation contributes to the killing of *M. tuberculosis* bacilli in mice treated with DSF. This activity could be in addition to a direct Cu-dependent killing mechanism on bacteria. The prospect of DSF having both a direct anti-bacterial effect and a ‘host-enabling' mechanism of action could be a strong justification to move DSF into the clinics for the treatment of TB.

## 6. Aldehydes beyond *Mycobacterium tuberculosis* infections?

The idea that bacteria encounter toxic aldehydes during infections is not new. McEwan and co-workers [24] noted that group A *Streptococcus* (GAS) encodes a glyoxylase (GloA) that is presumed to detoxify MG, yet does not encode a MG synthase. While MG can form spontaneously from products of glycolysis, the lack of an MG synthase in GAS nonetheless led to the McEwan and co-workers [24] to hypothesize that these bacteria use GloA to combat MG produced by neutrophils. Indeed, the authors of this work found that a GAS strain defective in *gloA* is more sensitive to MG and more susceptible to killing by human neutrophils *in vitro* than a parental strain. Furthermore, depletion of glucose from cell culture media reverses the sensitivity of a *gloA* mutant in these neutrophils, emphasizing the importance of glycolysis in the production of one or more toxic metabolites. More recently, Portnoy and co-workers [44] showed that mutants of *Listeria monocytogenes* lacking GloA are highly attenuated in mice. Because this phenotype is rescued by constitutive activation of PrfA, a bacterial transcription factor activated upon entry into host cell cytosol, the authors speculate that host-derived MG is a cue that bacteria sense to activate transcription of virulence genes. In addition, Woodward and co-workers [45] proposed *L. monocytogenes* encodes genes to deal with 4-HNE generated by ROS during infections. In this study, the authors looked for genes that were induced in the presence of 4-HNE, with the hypothesis that these genes encode detoxifying products of this lipid aldehyde encountered in animal tissues. Two enzymes, RhaA1 and RhaA2, were identified to have detoxification activity *in vitro*. Heterologous expression of these genes in *Bacillus subtilis*, a non-pathogenic bacterial species, provides some protection against 4-HNE *in vitro*. While this study showed *L. monocytogenes* infection induces 4-HNE in infected mouse tissues, the *rhaA* genes do not provide a fitness advantage to the parental strain in mice. It is possible that additional or different gene products are needed for 4-HNE resistance or that deletion of *rhaA* genes results in the induction of compensatory pathways to mitigate aldehyde toxicity *in vivo*. Another reason why no difference was observed could be that given that *L. monocytogenes* is generally not pathogenic to immunocompetent hosts and can be readily cleared, 4-HNE-sensitive mutants might not necessarily show fitness defects in immunocompetent infection models.

While the mechanism(s) of aldehyde toxicity have not been determined in any of the above studies, it is possible that aldehydes target numerous pathways, collectively inactivating bacterial growth or survival *in vivo*. This non-specific, ‘death by a thousand cuts’ feature of aldehydes may make it an ideal weapon against invading microbes.

## 7. Going forward

**Figure 1.**
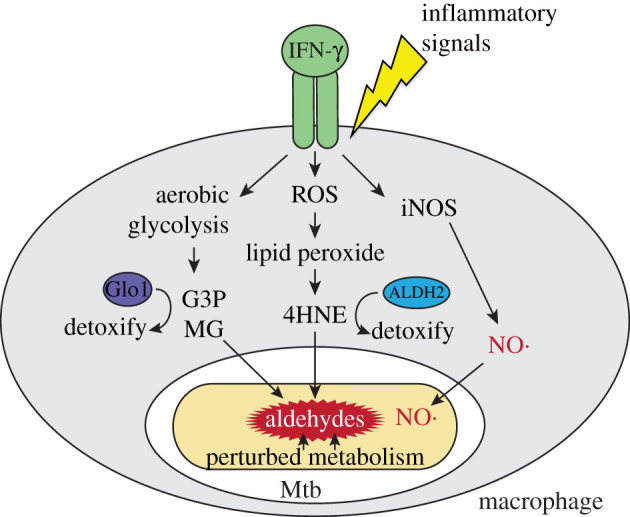
Model of infection control by metabolic aldehydes. In the context of inflammatory signals such as from pattern recognition receptors and IFN-γ, increased aldehyde intermediates accumulate from lipid peroxidation by ROS and increased glycolysis in macrophages. These molecules may synergize with NO or Cu to kill Mtb. Reduced expression of endogenous aldehyde detoxification genes (*ALDH2*, *GLO1*) due to inflammatory signals or a naturally occurring mutation in ALDH2 in humans or treatment with an FDA-approved inhibitor disulfiram could amplify aldehyde-mediated antimicrobial activity.

We propose a model whereby mammalian macrophages exploit aldehydes produced during inflammation to target invading pathogens (figure 1). How could this model be tested, given that testing the contributions of specific host cell effectors in inhibiting bacterial growth can be challenging? In some cases, proposed effectors are essential for normal cellular processes in the host. Other challenges manifest in the numerous limitations of cell culture infection models. For example, *in vitro* cell culture models of *M. tuberculosis* infection are generally limited to 6–10 d, after which host cells die. Given that *M. tuberculosis* infections can last for years and even decades, it is difficult to envision a week-long experiment modelling the reality of natural infection. Additionally, *in vitro* monoculture fails to reproduce the complex cellular interplay encountered *in vivo* (i.e. the presence of T cells is critical for the robust control of mycobacterial infections). While these and other reasons have made it challenging to quantify the relative contributions of different effectors, mouse infection models along with bacterial mutants with variable aldehyde susceptibility should make the aldehyde hypothesis testable.

## Data Availability

This article has no additional data.

## References

[1] Weiss G, Schaible UE. 2015 Macrophage defense mechanisms against intracellular bacteria. Immunol. Rev. **264**, 182-203. (10.1111/imr.12266) 25703560 PMC4368383

[2] Behr MA, Kaufmann E, Duffin J, Edelstein PH, Ramakrishnan L. 2021 Latent tuberculosis: two centuries of confusion. Am. J. Respir. Crit. Care. Med. **204**, 142-148. (10.1164/rccm.202011-4239PP) 33761302 PMC8650795

[3] WHO 2019 *Global tuberculosis report*. Geneva, Switzerland: World Health Organization.

[4] Flynn JL, Chan J, Triebold KJ, Dalton DK, Stewart TA, Bloom BR. 1993 An essential role for interferon gamma in resistance to *Mycobacterium tuberculosis* infection. J. Exp. Med. **178**, 2249-2254. (10.1084/jem.178.6.2249) 7504064 PMC2191274

[5] MacMicking JD, North RJ, LaCourse R, Mudgett JS, Shah SK, Nathan CF. 1997 Identification of nitric oxide synthase as a protective locus against tuberculosis. Proc. Natl Acad. Sci. USA **94**, 5243-5248. (10.1073/pnas.94.10.5243) 9144222 PMC24663

[6] Mishra BB et al. 2017 Nitric oxide prevents a pathogen-permissive granulocytic inflammation during tuberculosis. Nat. Microbiol. **2**, 17072. (10.1038/nmicrobiol.2017.72) 28504669 PMC5461879

[7] Mishra BB, Rathinam VA, Martens GW, Martinot AJ, Kornfeld H, Fitzgerald KA, Sassetti CM. 2013 Nitric oxide controls the immunopathology of tuberculosis by inhibiting NLRP3 inflammasome-dependent processing of IL-1β. Nat. Immunol. **14**, 52-60. (10.1038/ni.2474) 23160153 PMC3721324

[8] Wolschendorf F et al. 2011 Copper resistance is essential for virulence of *Mycobacterium tuberculosis*. Proc. Natl Acad. Sci. USA **108**, 1621-1626. (10.1073/pnas.1009261108) 21205886 PMC3029754

[9] Rowland JL, Niederweis M. 2012 Resistance mechanisms of *Mycobacterium tuberculosis* against phagosomal copper overload. Tuberc. (Edinb) **92**, 202-210. (10.1016/j.tube.2011.12.006) PMC3323751 22361385

[10] Shi X, Festa RA, Ioerger TR, Butler-Wu S, Sacchettini JC, Darwin KH, Samanovic MI. 2014 The copper-responsive RicR regulon contributes to *Mycobacterium tuberculosis* virulence. mBio **5**, e00876-13. (10.1128/mBio.00876-13) 24549843 PMC3944814

[11] Ward SK, Abomoelak B, Hoye EA, Steinberg H, Talaat AM. 2010 CtpV: a putative copper exporter required for full virulence of *Mycobacterium tuberculosis*. Mol. Microbiol. **77**, 1096-1110. (10.1111/j.1365-2958.2010.07273.x) 20624225 PMC2965804

[12] White C, Lee J, Kambe T, Fritsche K, Petris MJ. 2009 A role for the ATP7A copper-transporting ATPase in macrophage bactericidal activity. J. Biol. Chem. **284**, 33 949-33 956. (10.1074/jbc.M109.070201) PMC2797165 19808669

[13] Wagner D, Maser J, Lai B, Cai Z, Barry III CE, Zu Bentrup KH, Russell DG, Bermudez LE. 2005 Elemental analysis of *Mycobacterium avium*-, *Mycobacterium tuberculosis*-, and *Mycobacterium smegmatis*-containing phagosomes indicates pathogen-induced microenvironments within the host cell's endosomal system. J. Immunol. **174**, 1491-1500. (10.4049/jimmunol.174.3.1491) 15661908

[14] Pethe K et al. 2010 A chemical genetic screen in *Mycobacterium tuberculosis* identifies carbon-source-dependent growth inhibitors devoid of in vivo efficacy. Nat. Commun. **1**, 1-8. (10.1038/ncomms1060) 20975714 PMC3220188

[15] Safi H, Gopal P, Lingaraju S, Ma S, Levine C, Dartois V, Alland D. 2019 Phase variation in *Mycobacterium tuberculosis glpK* produces transiently heritable drug tolerance. Proc. Natl Acad. Sci. USA **116**, 19 665-19 674. (10.1073/pnas.1907631116) PMC6765255 31488707

[16] Bellerose MM et al. 2019 Common variants in the glycerol kinase gene reduce tuberculosis drug efficacy. mBio. **10**, e00663-19. (10.1128/mBio.00663-19) 31363023 PMC6667613

[17] Puckett S et al. 2017 Glyoxylate detoxification is an essential function of malate synthase required for carbon assimilation in *Mycobacterium tuberculosis*. Proc. Natl Acad. Sci. USA **114**, E2225-E2232. (10.1073/pnas.1617655114) 28265055 PMC5358392

[18] Maksymiuk C, Balakrishnan A, Bryk R, Rhee KY, Nathan CF. 2015 E1 of alpha-ketoglutarate dehydrogenase defends *Mycobacterium tuberculosis* against glutamate anaplerosis and nitroxidative stress. Proc. Natl Acad. Sci. USA **112**, E5834-E5843. (10.1073/pnas.1510932112) 26430237 PMC4629369

[19] Samanovic MI et al. 2015 Proteasomal control of cytokinin synthesis protects *Mycobacterium tuberculosis* against nitric oxide. Mol. Cell. **57**, 984-994. (10.1016/j.molcel.2015.01.024) 25728768 PMC4369403

[20] Hazen SL, Hsu FF, d'Avignon A, Heinecke JW. 1998 Human neutrophils employ myeloperoxidase to convert alpha-amino acids to a battery of reactive aldehydes: a pathway for aldehyde generation at sites of inflammation. Biochemistry **37**, 6864-6873. (10.1021/bi972449j) 9578573

[21] Braverman J, Sogi KM, Benjamin D, Nomura DK, Stanley SA. 2016 HIF-1α is an essential mediator of IFN-γ-dependent immunity to *Mycobacterium tuberculosis*. J. Immunol. **197**, 1287-1297. (10.4049/jimmunol.1600266) 27430718 PMC4976004

[22] Braverman J, Stanley SA. 2017 Nitric oxide modulates macrophage responses to *Mycobacterium tuberculosis* infection through activation of HIF-1α and repression of NF-κB. J. Immunol. **199**, 1805-1816. (10.4049/jimmunol.1700515) 28754681 PMC5568107

[23] Gleeson LE, Sheedy FJ, Palsson-McDermott EM, Triglia D, O'Leary SM, O'Sullivan MP, O'Neill LA, Keane J. 2016 Cutting edge: *Mycobacterium tuberculosis* induces aerobic glycolysis in human alveolar macrophages that is required for control of intracellular bacillary replication. J. Immunol. **196**, 2444-2449. (10.4049/jimmunol.1501612) 26873991

[24] Zhang MM, Ong CL, Walker MJ, McEwan AG. 2016 Defence against methylglyoxal in group A Streptococcus: a role for glyoxylase I in bacterial virulence and survival in neutrophils? Pathog. Dis. **74**, ftv122. (10.1093/femspd/ftv122) 26702634

[25] Escoll P et al. 2017 *Legionella pneumophila* modulates mitochondrial dynamics to trigger metabolic repurposing of infected macrophages. Cell Host Microbe. **22**, 302-316. (10.1016/j.chom.2017.07.020) 28867389

[26] Escoll P, Buchrieser C. 2019 Metabolic reprogramming: an innate cellular defence mechanism against intracellular bacteria? Curr. Opin. Immunol. **60**, 117-123. (10.1016/j.coi.2019.05.009) 31247377

[27] Fan J, Kamphorst JJ, Mathew R, Chung MK, White E, Shlomi T, Rabinowitz JD. 2013 Glutamine-driven oxidative phosphorylation is a major ATP source in transformed mammalian cells in both normoxia and hypoxia. Mol. Syst. Biol. **9**, 712. (10.1038/msb.2013.65) 24301801 PMC3882799

[28] Mazurek S, Boschek CB, Hugo F, Eigenbrodt E. 2005 Pyruvate kinase type M2 and its role in tumor growth and spreading. Semin. Cancer Biol. **15**, 300-308. (10.1016/j.semcancer.2005.04.009) 15908230

[29] Rachman H et al. 2006 Critical role of methylglyoxal and AGE in mycobacteria-induced macrophage apoptosis and activation. PLoS ONE **1**, e29. (10.1371/journal.pone.0000029) 17183656 PMC1762319

[30] Thornalley PJ, Langborg A, Minhas HS. 1999 Formation of glyoxal, methylglyoxal and 3-deoxyglucosone in the glycation of proteins by glucose. Biochem. J. **344**, 109-116. (10.1042/bj3440109) 10548540 PMC1220620

[31] Prantner D, Nallar S, Richard K, Spiegel D, Collins KD, Vogel SN. 2021 Classically activated mouse macrophages produce methylglyoxal that induces a TLR4- and RAGE-independent proinflammatory response. J. Leukoc. Biol. **109**, 605-619. (10.1002/JLB.3A0520-745RR) 32678947 PMC7855181

[32] Schmoch T, Uhle F, Siegler BH, Fleming T, Morgenstern J, Nawroth PP, Weigand MA, Brenner T. 2017 The glyoxalase system and methylglyoxal-derived carbonyl stress in sepsis: glycotoxic aspects of sepsis pathophysiology. Int. J. Mol. Sci. **18**, 657. (10.3390/ijms18030657) 28304355 PMC5372669

[33] Aki T, Funakoshi T, Noritake K, Unuma K, Uemura K. 2020 Extracellular glucose is crucially involved in the fate decision of LPS-stimulated RAW264.7 murine macrophage cells. Sci. Rep. **10**, 10581. (10.1038/s41598-020-67396-6) 32601294 PMC7324593

[34] Park SK, Park CS, Lee HS, Park KS, Park BL, Cheong HS, Shin HD. 2014 Functional polymorphism in aldehyde dehydrogenase-2 gene associated with risk of tuberculosis. BMC Med. Genet. **15**, 40. (10.1186/1471-2350-15-40) 24690209 PMC3975138

[35] Ragan EJ, Kleinman MB, Sweigart B, Gnatienko N, Parry CD, Horsburgh CR, LaValley MP, Myers B, Jacobson KR. 2020 The impact of alcohol use on tuberculosis treatment outcomes: a systematic review and meta-analysis. Int. J. Tuberc. Lung Dis. **24**, 73-82. (10.5588/ijtld.19.0080) 32005309 PMC7491444

[36] Luo HR, Wu GS, Pakstis AJ, Tong L, Oota H, Kidd KK, Zhang YP. 2009 Origin and dispersal of atypical aldehyde dehydrogenase ALDH2487Lys. Gene **435**, 96-103. (10.1016/j.gene.2008.12.021) 19393179

[37] Liu Q et al. 2018 China's tuberculosis epidemic stems from historical expansion of four strains of *Mycobacterium tuberculosis*. Nat. Ecol. Evol. **2**, 1982-1992. (10.1038/s41559-018-0680-6) 30397300 PMC6295914

[38] Merker M, Blin C, Mona S, Duforet-Frebourg N, Lecher S, Willery E, Wirth T. 2015 Evolutionary history and global spread of the *Mycobacterium tuberculosis* Beijing lineage. Nat Genet. **47**, 242-249. (10.1038/ng.3195) 25599400 PMC11044984

[39] He Y, Zhou C, Huang M, Tang C, Liu X, Yue Y, Liu D. 2020 Glyoxalase system: a systematic review of its biological activity, related-diseases, screening methods and small molecule regulators. Biomed. Pharmacother. **131**, 110663. (10.1016/j.biopha.2020.110663) 32858501

[40] Deitrich RA, Erwin VG. 1971 Mechanism of the inhibition of aldehyde dehydrogenase in vivo by disulfiram and diethyldithiocarbamate. Mol. Pharmacol. **7**, 301-307. 4328422

[41] Horita Y, Takii T, Yagi T, Ogawa K, Fujiwara N, Inagaki E, Onozaki K. 2012 Antitubercular activity of disulfiram, an antialcoholism drug, against multidrug- and extensively drug-resistant *Mycobacterium tuberculosis* isolates. Antimicrob. Agents Chemother. **56**, 4140-4145. (10.1128/AAC.06445-11) 22615274 PMC3421551

[42] Dalecki AG, Haeili M, Shah S, Speer A, Niederweis M, Kutsch O, Wolschendorf F. 2015 Disulfiram and copper ions kill *Mycobacterium tuberculosis* in a synergistic manner. Antimicrob. Agents Chemother. **59**, 4835-4844. (10.1128/AAC.00692-15) 26033731 PMC4505271

[43] Shen ML, Lipsky JJ, Naylor S. 2000 Role of disulfiram in the in vitro inhibition of rat liver mitochondrial aldehyde dehydrogenase. Biochem. Pharmacol. **60**, 947-953. (10.1016/S0006-2952(00)00435-4) 10974203

[44] Anaya-Sanchez A, Feng Y, Berude JC, Portnoy DA. 2021 Detoxification of methylglyoxal by the glyoxalase system is required for glutathione availability and virulence activation in *Listeria monocytogenes*. PLoS Pathog. **17**, e1009819. (10.1371/journal.ppat.1009819) 34407151 PMC8372916

[45] Tabakh H, McFarland AP, Thomason MK, Pollock AJ, Glover RC, Zaver SA, Woodward JJ. 2021 4-Hydroxy-2-nonenal antimicrobial toxicity is neutralized by an intracellular pathogen. Elife **10**, e59295. (10.7554/eLife.59295) 33955352 PMC8174450

